# Evaluation of HTLV-I and HCV Prevalence in Non-Hodgkin’s Lymphoma

**Published:** 2013-03

**Authors:** Maryam Rastin, Ali Reza Khoee, Nafiseh Tabasi, Akram Sheikh, Saeed Ziaolhagh, Elham Esmaeeli, Shahrzad Zamani, Mahdieh Khazaee, Mahmoud Mahmoudi

**Affiliations:** 1 Immunology Research Centre, BuAli Research Institute, School of Medicine, Mashhad University of Medical Sciences, Mashhad, Iran; 2 Department of Pathology, Imam Reza Hospital, School of Medicine, Mashhad University of Medical Sciences, Mashhad, Iran

**Keywords:** Human T Lymphotropic Virus, Hepatitis C virus, Non-Hodgkin’s lymphoma

## Abstract

***Objective(s):*** Non-Hodgkin’s lymphoma (NHL) is a lymphoproliferative malignancy in which cells undergo microscopic changes with unknown etiology, and risk factors such as age, sex, genetic and environmental factors are involved. The relationship between the presence of infectious agents and the development of lymphoproliferative diseases has been an interesting research topic. HTLV-I (Human T Cell Lymphotropic Virus Type-1) predisposes the infected individulas to opportunistic neoplasms and lymphoid malignancies. HCV (Hepatitis C Virus) the etiologic agent of hepatitis C, is hepatotropic, and long-term infection with HCV can continuously stimulate and expand lymphocyte clones, resulting in further transformation and finally aggressive malignancies.

***Materials and Methods:*** 54 tissue samples diagnosed with NHL were selected to be studied for the presence of HTLV-I and HCV viruses. DNA and RNA were extracted from samples, cDNA was synthesized and using specific primers presence of HTLV-I and HCV viruses were investigated by PCR and nested RT-PCR methods.

***Results:*** In 10 out of 54 (18.8%) samples (7 men and 3 women), HTLV-I was present, and 4 out of 54 (7.4%) samples (3 men and one woman) were positive for HCV.

***Conclusion:*** Based on our results, it is recommended that in patients with NHL, infection with HTLV-I and HCV viruses need to be screened.

## Introduction

Non-Hodgkin’s lymphoma (NHL) is a lymphoproliferative malignancy in which many microscopic changes is seen in the cells of patients, during which T or B lymphocytes undergo malignant transformation (1). The etiology for these changes is unknown, but the risk factors such as age, sex, genetic and environmental factors have been involved (2). The relationship between the presence of some infectious agents and development of lymphoproliferative diseases has been an interesting research topic for the researchers, since associations have been reported between the presence of HTLV-I, Helicobacter pylori, EBV, HIV and HCV with various types of lymphoma (3). 

HTLV-I (Human T Cell Lymphotropic Virus Type-1) is a retrovirus endemic in certain areas of the world, including in the north east of of Razavi Khorasan Province, Iran (4). This virus causes Adult T Cell Leukemia/Lymphoma among 2-4% of infected individuals, and is associated with lymphoid malignancies (5). HTLV-I predisposes the infected people to opportunistic neoplasms and lymphoid malignancies by severe suppression of their immune system. The mechanism of causing malignancy by HTLV-I virus is not clear, but it seems that the gene products of Tax region in virus enhance transcription of the genes controlling cell growth factors such as IL-2 through activation of a number of transcription factors like NF-kβ. This results in increased proliferation of T lymphocytes. In addition, Tax region reduces the expression level of some controlling genes such as β-Polymerase, thereby increasing chromosomal anomalies through increasing DNA transcription errors (1). Contact with HTLV-I virus in the first years of life, especially from mother to child through breastfeeding, is important in the occurrence of lymphoid malignancies.

HCV (Hepatitis C Virus) is the etiologic agent of hepatitis C. HVC is not only hepatotropic but also lymphotropic and sialotropic. In addition to liver, it can be found in lymph nodes, pancreas, adrenal gland, thyroid, spleen and bone marrow, and therefore it seems to be able to reproduce and replicate in extrahepatic sites (7). HCV infection is associated with a wide range of immunological disorders and extrahepatic diseases such as type II and III cryoglobulinemia, NHL, membranoproliferative glomerulonephritis, autoimmune thyroiditis, and so forth (8). The role of HCV in development of the above diseases is not clear, but long-term infection with HCV can continuously stimulate and expand lymphocyte clones resulting in further mutation and transformation and finally aggressive malignancies such as NHL. 

The protein in core region of HCV has shown to have severe transacting activity, activating certain oncogenes. HCV may also cause transformation of the infected cells like lymphoid cells. Recently, a specific receptor for HCV has been identified as CD81 on hepatocytes and lymphocytes, which binds protein E2 in env region of the virus (9). The immune system status of the infected individuals, proliferation rate and mutation in virus together with genetic and environmental factors influence the type of diseases caused by HCV, so infection with this virus can cause a range of diseases like infectious disease or malignant lymphoma such as NHL. 

## Materials and Methods

In this study, 54 tissue samples which were diagnosed with NHL during the past forty years in the department of pathology, Imam Reza Hospital, Mashhad University of Medical Sciences were selected. After microscopic examination of the tissue samples and confirmation of diagnosis by a pathologist, two 5-micron sections from each tissue sample were prepared in two separate sterile microtubes.

*DNA extraction from paraffin embedded tissues*

One ml octane was added to the tissue sections and incubated at room temperature for 30 min (the micro-tubes were vortexed every 5 min), and was finally centrifuged 5 min at 8000 rpm, and the supernatant was discarded (this was done twice). Then, 0.5 ml pure ethanol was added to the precipitate in the bottom of tubes and centrifuged for 5 min at 8000 rpm, and the supernatant was discarded (this process was performed 2 times). After that, 2-3 drops of acetone was added to the resulting precipitate and incubated at a temperature of 50°C for 5 min with open lid to evaporate ethanol. 100 µl of digestion buffer (0.5% Tween, 1mM EDTA, 5 mM Tris base) containing 200 μg/ml proteinase. K enzyme was added and incubated at 37°C for 24 hr to perform enzymatic digestion. The proteinase K enzyme was inactivated after 10 min at 95°C. The microtubes were finally centrifuged for 1 min at 8000 rpm, and the supernatant containing extracted DNA was kept in a sterile tube at -20°C for PCR. 

*RNA extraction from paraffin embedded tissues*

To extract RNA from Paraffin embedded tissues, deparaffinization and enzymatic digestion procedures were done as stated in the previous paragraph. To the obtained solution, containing intracellular nucleic acids, 1 ml TRIZOL (Roche) was added and kept at 4°C for 5 min. Then, 200 µl chloroform was added and vortexed for 15 sec, and again incubated at 4°C for 5 min and then centrifuged in 14000 rpm at 4°C. Three phases (layers) are formed in this step; the upper phase is aqueous and contains RNA. This phase was transferred to a sterile microtube, and an equal volume of isopropanol and 1:10 volume of 3M sodium acetate (pH=5.2) was added, kept on ice for 30 min and centrifuged for 10 min at 4°C in 12000 rpm. The supernatant was discarded, 1 ml 75% ethanol was added and put on ice for 10 min after vortexing, and centrifuged for 10 min in 8000 rpm. The supernatant was discarded and the precipitate was left in room temperature for 5-10 min to let the alcohol evaporate. Finally, 20 µl sterile DEPC treated water was added to the precipitate and put at 50°C for 5 min to dissolve RNA, and was used as a template for cDNA synthesis. 

*cDNA synthesis*

Due to the unstable nature of RNA, cDNA should be synthesized from it by reverse transcription enzyme as soon as possible, and be subject to PCR. For synthesis of cDNA, the required materials were purchased from Fermentase Company, and cDNA synthesis procedure was performed according to the manufacturer’s recommendations.

Five µg (about 9 µl) of extracted RNA was added in a microtube, 1 µl (20 uM) random hexamer primer was added and the volume reached 11ml using DEPC treated water. The microtube contents were mixed and incubated at 70°C for 10 min, and immediately were put on ice, then 4 µl of (5×) buffer, 0.5 µl RNase inhibitor , 2 µl dNTPs and 1.5 µl of DEPC treated water was added and placed at 25°C for 5 min. Then, 1 µl (40 units) of M-MuLV enzyme (reverse transcriptase) was added and was first put 10 min at 25°C and then 60 min at 42°C to synthesize cDNA. Finally, the microtube was incubated for 10 min at 70°C until M-MuLV enzyme was inactivated. The cDNA produced was stored in -20°C freezer until the testing time. The presence of the HCV was assessed using Nested RT-PCR, and presence of HTLV-I was evaluated by PCR. 

*PCR*

PCR was performed using specific primers designed for tax and LTR regions of HTLV-I and 5’-non coding (5NCR) region of HC. The specificity of primers was checked using the information provided in the gene bank by the blast software. 

For the detection of HTLV-I genome, PCR was performed with specific primers in 25 µl volume in 45 cycles by adding 1-5 μg DNA, 0.3 μM each primers, 4mM MgCl_2_, 200 μM dNTPs and 1 unit Taq DNA polymerase with the following primers: 

LT1: 5´-AAAAGCGTGGAGACAGTTCAGGAGG-3´,

LT2: 5´-TCGTATCCCGGACGAGCCCCCAA-3´, 

HT1: 5´-GGATACCCAGTCTACGTGT-3´, 

HT2: 5´-GAGCCGATAACGCGTCCATCG-3´

After amplification, a 451bp fragment was obtained for LTR, and a 158bp fragment for tax regions of HTLV-I virus.

**Figure 1:**
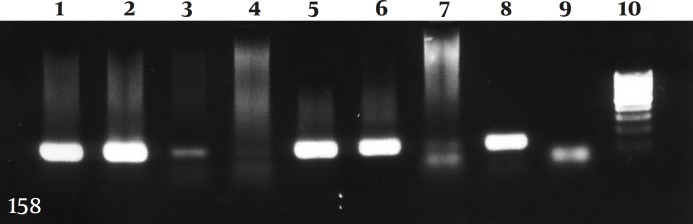
PCR for amplification of tax region of HTLV-I virus

Lanes 1-7: Patients’ samples, Lane 8: positive control, Lane 9: negative control, Lane 10: 100 bp DNA Ladder

Nested RT-PCR was performed with HCV specific primers in 25 μl volumes in the first step in 30 cycles by adding 5μg cDNA, 0.4 μM each primers and 2 mM MgCl_2_, 200 μM dNTPs and 1 unit Taq DNA polymerase, and the primers used in this step were as follows: 

HC1:5´-ACTGTCTTCACGCAGAAAGCGTCTAGCCAT-3´

HC2: 5´-CGAGACCTCCCGGGGCACTCGCAAGCACCC-3´ 

Second step PCR was done in 30 cycles with the addition of 10 μl DNA from the first step, 0.4 μM each primers, 2 mM MgCl_2_, 200 μM dNTPs and 1 unit Taq DNA pol using the following primers producing a 276bp product. 

HCV3: 5´- ACGCAGAAAGCGTCTAGCCATGGCGTTAGT-3´

 HCV4:5´- TCCCGGGGCACTCGCAAGCACCCTATCAGG-3´

After finishing PCR and RT-PCR, 5 μl of the products was electrophoresed in 2% agarose gel containing 0.3% ethidium bromide, and assessed using a transluminator. 

## Results

Based on the information extracted from the samples examined in this study, 22% of the samples were from gastrointestinal tumors, 62% from lymph nodes (75% from neck, and 25% from inguinal lymph nodes), and 16% from masses in other parts of the body (Table 1). 54% of patients were less than 50 years old and 46% were older than 50 years. 70% of the patients were male and 30% were female.

**Figure 2:**
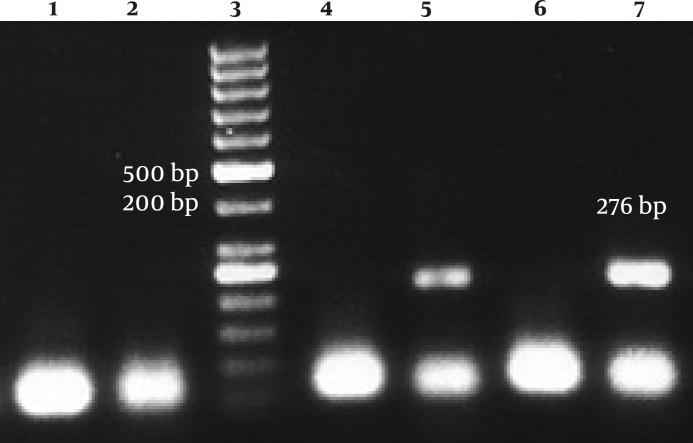
RT-PCR for HCV amplification

Lanes 1, 2, 4, 5: patients’ samples

Lane 6: negative control

Lane 7: positive control

Lane 3: 50bp DNA Ladder

The results of this study indicated that in 10 out of 54 (18.8%) NHL samples, the presence of HTLV-I was shown using specific primers for Tax, LTR and Pol regions of the virus. These cases included 7 men and 3 women, all had less than 50 years of age. HCV was positive in 4 out of 54 (7.4%) tissue samples using primers for the 5’-non coding region of HCV. These cases were 3 men and one woman, all were under 50 years old.

## Discussion

HTLV-I is a human retrovirus associated with different diseases like adult T cell leukemia/lymphoma (ATLL) and chronic neurological disorders including HTLV-I associated myelopathy/Tropical spastic paraparesis (HAM/TSP) (5). This virus weakens the immune system by infecting Th lymphocytes. The gene products of virus Tax region augment the transcription of controlling genes for cell growth by activating a number of transcription factors such as NF-kβ, resulting in increased proliferation of T lymphocytes (6). In addition, the expression level of some controlling genes such as β-polymerase is reduced, thereby increasing chromosomal anomalies through the increase in DNA transcription errors (1). The results of this study show that there is 18.8% correlation rate between NHL and the presence of HTLV-I, which is endemic in eastern regions of Iran. In a study performed on 32 patients in 1993 in Gabon, it was reported that HTLV-I was absent in 6 children with Burkitt’s lymphoma, while HTLV-I was present in 26.9% of the adult patients with NHL (10). In a study conducted in 2004 in Dominican Republic, the correlation rate of HTLV-I with hematologic malignancies and NHL was found to be 38.6% and 44.4%, respectively (11). In a study conducted in 1993 in Jamaica on 135 NHL patients, a high association rate of 63.3% was seen between HTLV-I presence and type T NHL (2), while in USA this association has been reported to be 4% (59). This difference in the rate of association between HTLV-I with non-Hodgkin’s lymphoma reported from different parts of the world seems to be due to the differences in the propagation of the virus in different regions. It seems that in endemic areas, there is a higher association with malignancies.

**Table 1:** Histological characteristics of NHL lymphoma samples in this study

		Place of resentment					
Histological forms of NHL	Number	GI Tract	Cervical lymphatic nodes	Inguinal lymphatic nodes	Other sites	HTLV-I positive cases	HCV positive cases
Disseminated	32	9	10	3	10	8	3
Nodular	6	unidentified	unidentified	unidentified	unidentified	-	-
Great cell lymphomas	5	-	4	-	1	-	-
Low grade lymphomas	2	-	-	1	1	-	1
Burkitt’s like lymphoma	1	-	1	-	-	1	-
Atypical NHL	2	-	-	-	2	-	-
NHL with mixed cellularity	5	-	3	-	2	1	-
Total	54	9	18	4	16	10	4

There are also several reports on the association between HCV infection and NHL, various researchers having reported different association rates. In a study conducted in Turkey in 2003, it has been stated that HCV is lymphotropic and sialotropic in addition to being hepatotropic, and its infection causes lymphotropic disorders with a reported 7.1% association rate with NHL (7). In another study in 2002, an association has been reported between HCV and NHL, cryoglobulinemia, membranous proliferative glomerulonephritis and Porphyria Cutanea Tarda (PCT).Those afflicted with these diseases were recommended to be screened for HCV infection (8). In a study in 1998, HCV replication in extrahepatic sites has been suggested, and HCV has been isolated from the liver, lymph nodes, pancreas, adrenal glands, thyroid, spleen and BM of infected people (12). In a study conducted in Brazil in 2002, the presence of HCV in patients with NHL was reviewed, and an association rate of 9% was reported with NHL (3). In our study, HCV was present in 7.4% of NHL patients. In a study conducted in 2004 in Spain, an association rate of 7% was also reported between HCV and non-Hodgkin’s lymphoma (13). In a study in California, 4.2% of NHL cases were sero positive for HCV, but in none of them RNA of the virus was detected (14). 

These differences can be due to the environmental factors, race, and the presence of different genotypes, mutations or other factors. In most cases, genotype 2 of HCV has been detected in lymphoproliferative diseases, genotype 1b has been found in a few cases, and genotype 1a has not been found at all. Therefore, it could be concluded that perhaps the type of genotype is involved in the disease type following HCV infection. It seems that due to the differences in the genotypes prevalence in different regions, there are different association rates of HCV with malignancies. 

## Conclusion

In this study, we showed that the prevalence of HTLV-I virus, in this endemic in the northeast of Iran, and the prevalence of HCV virus have increased in NHL samples.
